# A feasibility trial to examine the social norms approach for the prevention and reduction of licit and illicit drug use in European University and college students

**DOI:** 10.1186/1471-2458-12-882

**Published:** 2012-10-18

**Authors:** Claudia R Pischke, Hajo Zeeb, Guido van Hal, Bart Vriesacker, John McAlaney, Bridgette M Bewick, Yildiz Akvardar, Francisco Guillén-Grima, Olga Orosova, Ferdinand Salonna, Ondrej Kalina, Christiane Stock, Stefanie M Helmer, Rafael T Mikolajczyk

**Affiliations:** 1 BIPS - Institute for Epidemiology and Prevention Research, Achterstraße 30, 28359, Bremen, Germany; 2 Faculty of Medicine and Health Sciences, University of Antwerp, Antwerp, Belgium; 3 Division of Psychology, University of Bradford, Bradford, UK; 4 Leeds Institute of Health Sciences, University of Leeds, Leeds, UK; 5 School of Medicine, Marmara University, Istanbul, Turkey; 6 Department of Health Sciences, Public University of Navarra, Navarra, Spain; 7 Department of Educational Psychology and Health Psychology, PJ Safaric University, Kosice, Slovakia; 8 Institute of Public Health, University of Southern Denmark, Esbjerg, Denmark; 9 Helmholtz Centre for Infection Research, Braunschweig, Germany; 10 Hannover Medical School, Hannover, Germany

**Keywords:** Social norms, Prevention, Drug use, Intervention, University/College students, Europe

## Abstract

**Background:**

Incorrect perceptions of high rates of peer alcohol and tobacco use are predictive of increased personal use in student populations. Correcting misperceptions by providing feedback has been shown to be an effective intervention for reducing licit drug use. It is currently unknown if social norms interventions are effective in preventing and reducing illicit drug use in European students. The purpose of this paper is to describe the design of a multi-site cluster controlled trial of a web-based social norms intervention aimed at reducing licit and preventing illicit drug use in European university students.

**Methods/Design:**

An online questionnaire to assess rates of drug use will be developed and translated based on existing social norms surveys. Students from sixteen universities in seven participating European countries will be invited to complete the questionnaire. Both intervention and control sites will be chosen by convenience. In each country, the intervention site will be the university that the local principal investigator is affiliated with. We aim to recruit 1000 students per site (baseline assessment). All participants will complete the online questionnaire at baseline. Baseline data will be used to develop social norms messages that will be included in a web-based intervention. The intervention group will receive individualized social norms feedback. The website will remain online during the following 5 months. After five months, a second survey will be conducted and effects of the intervention on social norms and drug use will be measured in comparison to the control site.

**Discussion:**

This project is the first cross-national European collaboration to investigate the feasibility of a social norms intervention to reduce licit and prevent illicit drug use among European university students.

**Final trial registration number:**

DRKS00004375 on the ‘German Clinical Trials Register’.

## Background

Licit and illicit drug use remains a major public health threat in Europe. One quarter of European 18–21 year olds and 41% of 21–24 years olds report having consumed an illicit drug (i.e., cannabis, amphetamines, ecstasy, LSD, opiates, cocaine, crack or mushrooms) in their lifetime [1]. Four percent of all European Union (EU) deaths among those aged 15–39 years are drug-related [1]. The harmful use of legal drugs, such as tobacco and alcohol, also continues to be a problem in the EU. For example, in Germany, 21% of young adults report binge drinking at least once a month and 30% of women and 38% of men aged 20–24 are regular smokers [2-6]. Lastly, the use of multiple drugs at the same time (i.e., polydrug use) is widespread in Europe with the majority of drug use combinations including alcohol [1]. To date, no large-scale study has compared single and polydrug use by college and university students living in different EU countries.

Public health strategies and policies addressing issues of drug use in Europe are heterogeneous. Some European countries have a strategy for preventing illicit drug use but none for alcohol whereas others have separate or interlinked strategies for illicit drugs and alcohol compared to yet others without a national policy regarding drug use [1]. There are multiple reasons for the absence of a shared European policy model addressing issues of drug use across all European countries. One reason is that prevalence rates of short- and evidence of long-term health consequences associated with licit and illicit drug use vary by country [1,7-9] and pose differential demands on the respective national health care systems (which also vary by European country). Secondly, legal ramifications of licit and illicit drug use vary across countries. This diversity renders a development of a joint public health policy to address drug use among young European adults problematic. Instead of focusing on the development of such a joint policy, public health strategies aimed at changing social and interpersonal processes surrounding drug use among young adults may be more feasible for the prevention and reduction of drug use.

Social influence in the form of social norms, or the “perceptions and beliefs what is ‘normal’ behaviour in the people close to us” (p.3, [10]) has been identified as a key factor modifying drug use behaviour among young adults [11-13]. It is known that individuals, and young adults in particular, tend to overestimate drug use in their respective peer group and that these incorrect perceptions are predictive of higher rates of personal drug use [14-20]. In regard to alcohol use, these misperceptions can be about both rates of peer alcohol use (***descriptive norms***) and the social acceptability of alcohol use (***injunctive norms***). Individuals may overestimate the frequency and quantity of alcohol consumption of their peers, and also overestimate how acceptable their peers feel heavy drinking to be. The individual is then motivated to match their own alcohol consumption to what is an incorrect perception [21,22]. A smaller number of studies have evaluated the role of injunctive norms on illicit drug use. For example, one study showed that students tend to overestimate the level of approval of marijuana use behaviour in their peer group [23].

The social norms approach is one harm reduction strategy that has gained rapid recognition in the past two decades. This approach takes advantage of young adults’ susceptibility to peer influence. The approach works on the premise that if misperceptions are challenged then the social pressure on the individual will lessen and their own rate of use will fall. In the case of alcohol consumption, a social norms campaign may consist of surveying a college student population to identify the actual and perceived rates of alcohol use, and then presenting this information back to the student population. Traditional social norms campaigns have done this by providing social norms feedback to student populations through mass media campaigns and a variety of peer education activities. This approach has been found to be an effective method of reducing alcohol and drug harm at several college campuses [24,25], and has also been used successfully to address other risky behaviours [26]. More recently, online technology has been used to offer individuals personalised social norms feedback. Online feedback operates on the same principles as mass media social norms campaigns, except that the discrepancy between personal consumption, perceived peer consumption and actual reported peer consumption is made even more explicit to the individual. Preliminary research suggests that instantaneous, personalised, computer delivered feedback can be highly effective [27]. There is, however, a relative paucity of empirical studies which have explored this technique.

The social norms approach originated in the United States of America (USA) and to date many of the published studies address reduction of alcohol and drug harm on American college campuses. Initial studies assessing rates of drug use and associated social norms in European students indicate that a discrepancy between perceived and actual social norms on tobacco and alcohol use also exists in European young adult and student populations [18-20,28]. This raises the possibility of using the social norms approach to address risky health behaviours in Europe in the same way that it has been used in the USA. A relatively small number of social norms campaigns have been implemented in Europe and Australia [26]. Limitations in the existing evidence base mean that there is however a need for further studies investigating the feasibility of using this approach outside of the USA. In particular, there are several cultural and legislative differences between the USA and European countries that could potentially moderate both the role of misperceptions in alcohol and drug use behaviour, as well as the outcome of a social norms campaign. In addition, there are several gaps in the literature which need to be addressed. Firstly, there is a need to more fully explore the potential of online personalised feedback social norms campaigns in university and college settings. Secondly, there is a lack of research on the social norms approach in the prevention of tobacco, illicit and polydrug use, which is identified as an area of action in the EU Drug Action Plan 2009 – 2012. Finally, there is a lack of multi-language social norms interventions which can be applied simultaneously to students in different countries. If the social norms approach is to be implemented in more culturally and geographically diverse settings such as Europe then it is important these issues are addressed.

The objective of this paper is to describe the aims and study design of the project, entitled ‘Social Norms Intervention for the prevention of Polydrug usE (SNIPE)’. SNIPE is a European co-operation project funded by the European Commission, Directorate General Justice, Freedom and Security. This paper will outline the SNIPE project, a multi-site cluster controlled trial of a web-based social norms intervention aimed at reducing licit and preventing illicit and polydrug use in university and college students in seven participating countries. SNIPE is the first cross-national European study investigating the feasibility of such an intervention.

## Methods/Design

### Aims of the project

This project aims to examine the feasibility of an intervention to prevent and reduce the consumption of licit and illicit drugs among university students in six European countries and Turkey, which is a candidate country for the EU (for simplicity we refer to seven European countries in the remaining text). The specific aims of the SNIPE project are:

a) To assess and compare self-reported consumption rates of licit and illicit drugs among university and college students from at least two universities or colleges in seven European countries

b) To examine the feasibility (i.e., understanding, utility and applicability) of a web-based social norms intervention in the participating countries, and

c) To compare the effects of this e-health intervention on related norms and consumption of both licit (alcohol, especially binge drinking, tobacco and sedatives) and illicit drug use (cannabis, cocaine, synthetic drugs, not prescribed medication, inhalants) in study participants allocated to the intervention with a control group over the course of 5 months.

### Study design

The SNIPE study is a multi-site cluster controlled trial conducted in seven European countries. Each country aims to recruit 2000 students at two or more different universities (or colleges): n=1000 at the university serving as the intervention site, n=1000 at a second university serving as the control site. Both intervention and control sites will be chosen by convenience. In each country, the intervention site will be the university that the local principal investigator is affiliated with. The total duration of the project will be 24 months. Data collection instruments and preliminary work on the intervention will take place over the summer preceding the start of the academic year. Data will then be collected from students at both intervention and control sites during the start of autumn semester of the academic year (T0). This information will then be used as the basis for the online intervention, which will be made available to students at the intervention sites later in the semester. Discussion groups will be held with students during the development and implementation of the intervention and changes may be made according to their input. A second period of data collection will take place towards the end of the spring semester of the same academic year at both intervention and control sites (T1). At the end of the study, students at the control sites will be given access to the intervention.

### Sample size calculation

For the purpose of describing relevant social norms and behaviours, we aim to reach a sample size of 1000 participants in the baseline surveys, allowing the estimation of prevalence with a 95% confidence interval of max. +/− 3%. Assuming a 40% loss during follow-up, 600 students are expected to participate in the second survey at each site of each participating country. This sample size is sufficient to detect a difference in the rate of binge drinking between the intervention and control sites (at follow-up) corresponding to an effect size of 0.2 at the level of p≤.05 with 89% power. The standardized effect size of 0.2 was reported for a binge drinking reduction in a previous study [27] and is a weak effect according to Cohen. This sample size calculation is based on the assumption of a relatively low intra-cluster correlation (0.02), i.e., assuming small differences between countries.

### Ethics

The study protocol was approved by the relevant institutional review boards or ethics committees in all participating countries (i.e., University of Bremen, Bremen, Germany; University Hospital Antwerp and the University of Antwerp, Antwerp, Belgium; University of Bradford, Bradford, United Kingdom; Public University of Navarra, Navarra, Spain; University of Southern Denmark, Esbjerg, Denmark; University of Pavol Jozef Šafárik University, Košice, Slovak Republic; Marmara University School of Medicine, Istanbul, Turkey). SNIPE researchers obtained permission from deans of the respective universities/colleges in each country to recruit students at their universities.

### Setting and participants

Students from all faculties of the respective university or colleges and from all semesters will be invited to enrol in this study.

### Recruitment

Students will be contacted via email, the universities’ intranet or website, or via direct face-to-face communication in seminars. To increase the visibility of the study and to facilitate recruitment, flyers and postcards advertising the study will be printed and laid out at all participating universities and at communal areas around the university (information desks, cafeterias). The project will also be publicized in general local newspaper articles, in student newsletters, at local radio broadcasts, at university announcements, and at university lectures and seminars. Furthermore, information about the study will be provided on social media accounts such as Twitter and Facebook. Participants will be consented to the study upon their online-registration.

### Study registration

Students at both intervention and control sites will be invited to register on the project website, whilst the online survey is under development. When registering on the website they will only be asked to supply their email address. They will be told that by doing so, they will later be invited to take part in a project that will let them see how their alcohol and drug use compares to their peers. When the survey goes online all pre-registered students will be emailed a link to it and will be invited to take part. Simultaneously, efforts will be made at each site to advertise the website to students who have not already registered.

### Data collection

When these students log onto the website they will be asked to provide their email address and then proceed to the baseline survey. The baseline survey will include questions regarding the frequency of personal and peer drug use and related social norms. After a month students at the intervention site who will have completed the baseline survey will be emailed and notified that they can now access the intervention website.

All data collected up until this point therefore will become the baseline data. However, the intervention website will operate by asking students for their current use and perceptions so that this information can be immediately presented back to them alongside the actual campus norms. As such, although the baseline data will already be complete, the website will continue to collect data every time a student logs on to get their personalised feedback – i.e., every time the student wants to get personalised feedback they will have to do the survey again. New students who did not complete the survey during the baseline period can still register, complete the survey, and get their personalised feedback. The second data collection phase will occur at 5 months. All study participants will be emailed and asked to visit the website to complete the survey, even if in the case of students at the intervention sites, they have already been visiting the website and completing the survey.

Students at both control and intervention sites will be given the same basic information – that by taking part in the project and completing the surveys they will be able to access personalised feedback. The only difference is that intervention site students will be told they will get access to this feedback in a short time period, whereas control site students will be told that they will get access the following year, i.e., the control group will receive access to this feedback after the follow-up assessment is completed.

### The social norms intervention

The social norms intervention will be an instantaneous personalized feedback and will take the following form: the perceived peer drug use (e.g., 60% of the male/female students at your university think that the majority of male/female students use marijuana at least once a month) will be contrasted with the assessed peer drug use among students of the same gender from the baseline questionnaire (e.g., 4% of the male/female students at your university use marijuana) to highlight discrepancies. Additionally, the personal drug use pattern (e.g., “I have five alcoholic drinks during a typical drinking session.”) will be put into relation to the drug use in the peer group (same-gender, same university, e.g., “Actually, most male students of my university (68%) drink no more than four alcoholic drinks during a typical drinking session!”). These two comparisons will form the ***descriptive norms*** feedback. In addition, information on ***injunctive norms*** (i.e., general perceptions about whether drug use is accepted in the peer group) will be provided in some of the feedback messages (e.g., “Did you know that 91% of male students at Bradford think it is never okay to use ecstacy?”).

Study participants from the intervention sites will be invited to access the feedback approximately two weeks after the baseline assessment and they will be informed that they will have the opportunity to access the intervention multiple times during the next 5 months. It is important to note that every time a student wishes to get feedback using the intervention they will have to first provide information about their own drug behaviour and perceptions.

All intervention materials including the text for the registration page, the baseline screening survey, and the feedback will be developed in English. The English version will then be translated into Dutch, Danish, German, Slovakian, Turkish, and Spanish. All materials will be pre-tested with students in each country.

### Content of the questionnaire

#### Demographic information

The questionnaire will include demographic questions on age, gender, religiosity, place of residence (e.g., university accommodation with other students, private accommodation), disposable income, disposable income spent on alcohol, tobacco and other drugs, country of origin, length of stay in the respective country and whether a student came to study to their current country. Participants will also be asked to provide information regarding their degree, subject, and year of their study.

#### Drug use

The use of the following drugs will be assessed: Alcoholic beverages (beer, wine, spirits, etc.), tobacco products (cigarettes, chewing tobacco, cigars, etc.), cannabis (marijuana, pot, grass, hash, etc.), medication to improve academic performance (Ritalin) which was not prescribed, synthetic cannabis (spice, etc.), cocaine (coke, crack, etc.), ecstasy, other amphetamine-type stimulants (speed, meth, etc.), sedatives or sleeping pills (diazepam, alprazolam, flunitrazepam, midazolam, stilnoct, etc.) which were not prescribed, hallucinogens (LSD, acid, mushrooms, trips, ketamine, etc.), inhalants (nitrous, glue, petrol, paint thinner, etc.). In addition, binge drinking and polydrug use (alcohol and tobacco, alcohol and any other illicit drug) will be assessed. The choice of drugs included will be based on the Alcohol, Smoking and Substance Involvement Screening Test (ASSIST), developed by the World Health Organisation [29].

A number of revisions will be made to adapt this measure for use in a student population. An item on the use of non-prescribed medication used as either sedatives or to improve academic performance will be included, in light of the existing literature on this issue [30]. In contrast to the ASSIST measure, separate items will be used to measure ecstasy use as opposed to other amphetamine type stimulants. Recent research suggests that after several years of declining use, there has been a recent resurgence in the use of ecstasy in young adult populations [31]. Therefore it is of interest to measure use of this substance separately so that more precise data on rates of use in student populations can be determined. This also allows for specific norms messages on ecstasy to be delivered to students during the intervention. The item on the use of opioids in the ASSIST will not be used in the current study, as previous work would suggest that use of this drug is relatively low in young adults populations [31] and there is a need to keep the survey to be used in this study at an overall length which participants can realistically be expected to complete.

The response options that will be used for the substance use items will follow the same pattern of ascending frequency as similar surveys in the area, such as the CORE Alcohol and Drug Survey Long Form [32], which is delivered annually to college students in the USA. The first response option is ‘Never in my life’ followed by ‘Have used but not in the last two months’, ‘Once in the last two months’, ‘Twice in the last two months’ and so on up to ‘Every day or nearly every day’. The time frame of the previous two months will cover the period when students are attending university, as planned by the schedule of data collection.

#### Perceptions of rates of peer drug use

Perceptions of rates of peer drug use will be assessed using items based on the corresponding personal use items. As each social norms survey is by necessity specific to the target population being studied, there are not any single existing measures which can be used for the current project. However, the perceptions items which have been designed will follow the same principle of previous social norms surveys [18,33], in which the perception item will be as closely worded to the personal use item as possible.

#### Attitudes toward personal and peer drug use

Personal and perceived social norms regarding attitudes towards drug use will also be assessed. These items will again be based on existing research [32] and will be tailored to the target population of students. The response options range from ‘Never ok to use’ to ‘Ok to use frequently if that is what the person wants to do‘.

#### Frequency of negative consequences in relation to drug use

Items based on the CORE Alcohol and Drug Survey Long Form [32] will be used to assess negative consequences of getting drunk and of other drug use (e.g., missing a class or another commitment, unprotected sex, engagement in violent acts).

### Analysis of data

#### Baseline analyses

Baseline descriptive analyses of social norms and drug use behaviour by gender, social class, nationality, religiosity and residence (e.g., living with parents or other students) will be conducted. Detailed analyses on rates of drug use between reported and perceived drug use and the factors which predict these will be conducted using a MANCOVA analysis.

#### Outcome evaluation of the intervention trial

Individual changes in drug use behaviours and social norms between baseline and follow-up in intervention and control sites will be tested by bivariate tests and in adjusted regression analyses, similar to previous work [34]. In addition, potential dose response relationships between frequency of intervention use (e.g., # of times the feedback was accessed online) and changes in norms and behaviours will be evaluated to quantify the “minimal dose” of the intervention or feedback received necessary to produce changes. In further steps, structural equation models will be used to assess relationships between changes in norms and drug use behaviours [35,36].

#### Process evaluation

To document the process of conducting the current study, a process evaluation will be conducted that focuses on recruitment, data collection, intervention development and implementation. Information will be collected throughout the research study. Data collection will primarily be via completion of bespoke written questionnaires by research leads in each country. Email correspondence pertaining to the process evaluation will also be routinely collected and included as a secondary source of data.

## Discussion

In the past three decades, a multitude of public health strategies targeted toward the prevention of drug use among young adults emerged in the USA and the EU. Some involved anti-drug media campaigns aimed at informing about harmful health consequences of licit and illicit drugs, such as the “Drugwatch” campaign in the USA. Others were educational interventions for drug use prevention informing about the harmful effects of drug use at schools and universities [37,38]. The majority of these prevention approaches were ineffective in reducing rates of licit and/or illicit drug use in young adults [26,39].

Major shortcomings of these approaches included the use of fear appeals and scare tactics, which often emphasize the harmful effects of drugs. These messages may not be taken seriously by the target population because negative consequences of drug use are often overstated and students often correctly perceive that the majority of these consequences are unlikely to occur [39]. In addition, some of the earlier anti-drug campaigns were based on the “Social Inoculation Theory”, according to which teaching students skills to resist peer pressures or “inoculating” them against social influences to use drugs will prevent actual drug use [40]. Our study aims to examine an alternative approach, the social norms approach, which acknowledges the influence of peers on young adults’ drug use behaviour and the role of social norms surrounding drug use in the peer group. Instead of inoculating students against social influence of their peers, this influence is leveraged to affect students’ drug use behaviour by correcting exaggerated perceptions of risky behaviours in the peer group.

The SNIPE project is the first cross-national European multisite cluster-controlled trial to assess and reduce and/or prevent the consumption of licit and illicit drugs among university and college students using the social norms approach. The three major innovations in this study are the application of the social norms approach to the realm of illicit and polydrug use; the comparison of rates of drug use and social norms across the participating countries and the study of feasibility of the same social norms intervention in multiple countries at the same time.

Because this intervention is implemented online, it can be easily made available to other student populations across Europe. We will attempt to disseminate the intervention, should we be able to demonstrate that it is feasible in the European context. A subsequent study in additional European countries and including a longer follow-up is conceivable.

Future avenues of social norms research may also include the conception of studies investigating why and how a person chooses a certain group as a social referent and how they perceive the behaviour of these groups. To date, research has focussed on students’ perceptions of other students on their campus. However, there is a lack of work exploring misperceptions in smaller sub-groups of peers. For example, a student’s specific perceptions of the behaviours of the peers in their class rather than just their perception of other students on the campus overall. Further insights into these issues may help us effectively tailor future social norms interventions to persons belonging to various peer groups and to compare intervention effects across peer groups varying in socio-demographic characteristics.

To conclude, the SNIPE study will provide data on rates of drug use and on perceptions about the consumption of licit and illicit drugs among university and college students comparing seven European countries. Further, it will provide answers toward the feasibility of a social norms intervention designed for the reduction of licit and the prevention of illicit drug use at institutions of higher education in the European context.

## Competing interests

The authors declare that they have no competing interests. In the past Bewick has received funding from the European Research Advisory Board and has received travel expenses from Anheuser-Busch.

## Authors’ contributions

All authors contributed to the conception and design of the study, critically revised the manuscript and approved the final document of this protocol. No other manuscripts are under submission with other journals based on this study protocol.

## Pre-publication history

The pre-publication history for this paper can be accessed here:

http://www.biomedcentral.com/1471-2458/12/882/prepub

## References

[B1] European Monitoring Centre for Drugs and Drug Addiction The state of the drugs problem in Europe 2010 Annual Report, 2010 http://www.kom.gov.tr/Tr/Dosyalar/EMCDDA_rapor_2010_en.pdf

[B2] Statistisches Bundesamt Leben in Deutschland. Haushalte, Familien und Gesundheit - Ergebnisse des Mikrozensus http://www.destatis.de/jetspeed/portal/cms/Sites/destatis/Internet/DE/Presse/pk/2006/Mikrozensus/Pressebroschuere,property=file.pdf

[B3] Sebena R Orosova O Mikolajczyk RT van Dijk JP Selected socio-demographic factors and differences in patterns of alcohol use among university students BMC Public Healthin press 10.1186/1471-2458-11-849 PMC3282827 22067135

[B4] Akmatov MK Mikolajczyk RT Meier S Krämer A Alcohol consumption among university students in North Rhine-Westphalia, Germany-Results from a Multicenter cross-sectional study J Am Coll Health 2011 59 7 620 626 10.1080/07448481.2010.520176 21823957

[B5] Helmer S Mikolajczyk R Meier S Krämer A Drogenkonsum von Studierenden – Ergebnisse des Gesundheitssurveys NRW Public Health Forum 2010 18 2 e1 e21

[B6] Stock C Mikolajczyk R Bloomfield K Maxwell AE Ozcebe H Petkeviciene J Naydenova V Marin-Fernandez B El-Ansari W Kramer A Alcohol consumption and attitudes towards banning alcohol sales on campus among European university students Public Health 2009 123 2 122 129 10.1016/j.puhe.2008.12.009 19185890

[B7] Center for Disease Control Persons who use drugs. Infections among persons who use drugs http://www.cdc.gov/pwud/

[B8] World Health Organisation Management of substance abuse. Facts and Figures http://www.who.int/substance_abuse/facts/en/

[B9] Medline Plus. A Service of the U.S.: National Library of Medicine. NIH National Institutes of Health Drug dependence http://www.nlm.nih.gov/medlineplus/ency/article/001522.htm

[B10] Moreira MT Smith LA Foxcroft D Social norms interventions to reduce alcohol misuse in university or college students Cochrane database of systematic reviews (Online) 2009 H. 3, S. CD006748 10.1002/14651858.CD006748.pub2 19588402

[B11] Berkowitz AD An overview of the social norms approach Changing the culture of college drinking: A socially situated health communication campaign 2005 Hampton Press, NJ

[B12] Perkins HW Perkins HW The emergence and evolution of the social norms approach to substance abuse prevention The social norms approach to preventing school and college age substance abuse: a handbook for educators, counselors, and clinicians 2003 Jossey-Bass, San Francisco 3 17

[B13] Perkins HW Meilman PW Leichliter JS Cashin JR Presley CA Misperceptions of the norms for the frequency of alcohol and other drug use on college campuses J Am Coll Health 1999 47 6 253 258 10.1080/07448489909595656 10368559

[B14] Haines MP Barker G Perkins HW The NIU experiment: A case study of the social norms approach The social norms approach to preventing school and college age substance abuse: A handbook for educators, counselors, and clinicians 2003 Jossey-Bass, San Francisco, CA 21 34

[B15] Perkins HW Craig DW Perkins HW The Hobart and William Smith College experiment: A synergistic social norms approach using print, electronic media, and curriculum infusion to reduce collegiate problem drinking Social norms approach to preventing school and college age substance abuse: A handbook for educators, counselors, and clinicians 2003 Jossey-Bass, San Francisco, CA 35 46

[B16] Johannessen K Glider P Perkins HW The University of Arizona's campus health social norms media campaign The social norms approach to preventing school and college age substance abuse: A handbook for educators, counselors, and clinicians 2003 Jossey-Bass, San Francisco 65 82

[B17] Kilmer JR Walker DD Lee CM Palmer RS Mallett KA Fabiano P Larimer ME Misperceptions of college student marijuana use: implications for prevention J Stud Alcohol 2006 67 2 277 281 16562410 10.15288/jsa.2006.67.277

[B18] McAlaney J McMahon J Normative beliefs misperceptions and heavy episodic drinking in a British student sample J Stud Alcohol Drugs 2007 68 385 392 17446978 10.15288/jsad.2007.68.385

[B19] Bewick BM Trusler K Mulhern B Barkman M Hill AJ The feasibility and effectiveness of a web-based personalised feedback and social norms alcohol intervention in UK university students: A randomised controlled trial Addict Behav 2008 33 1192 1198 10.1016/j.addbeh.2008.05.002 18554819

[B20] Page RM Ihasz F Hantiu I Simorek J Klarova R Social normative perceptions of alcohol use and episodic heavy drinking among Central and Eastern European adolescents Subst Use Misuse 2008 43 361 373 10.1080/10826080701202866 18365937

[B21] Festinger L A theory of social comparison processes Human Communications 1954 7 117 140

[B22] Bosari B Carey KB Peer influences of college drinking: A review of the research J Subst Abuse 2001 13 391 424 10.1016/S0899-3289(01)00098-0 11775073

[B23] LaBrie JW Hummer JF Lac A Lee CM Direct and indirect effects of injunctive norms on marijuana use: The role of reference groups J Stud Alcohol Drugs 2010 71 6 904 908 20946748 10.15288/jsad.2010.71.904 PMC2965299

[B24] Moreira MT Foxcroft DR The effectiveness of brief personalized normative feedback in reducing alcohol-related problems amongst university students: protocol for a randomized controlled trial BMC Public Health 2008 8 113 10.1186/1471-2458-8-113 18402657 PMC2322979

[B25] Turner J Perkins HW Bauerle J Declining negative consequences related to alcohol misuse among students exposed to a social norms marketing intervention on a college campus J Am Coll Health 2008 57 1 85 93 10.3200/JACH.57.1.85-94 18682350

[B26] McAlaney J Bewick B Hughes C The international development of the ‘Social Norms’ approach to drug education and prevention Drugs: Education Prevention and Policy 2011 18 2 81 89 10.3109/09687631003610977

[B27] Neighbors C Larimer ME Lewis MA Targeting misperceptions of descriptive drinking norms: Efficacy of a computer delivered personalised normative feedback intervention J Consult Clin Psychol 2004 72 3 434 447 15279527 10.1037/0022-006X.72.3.434

[B28] Lintonen T Konu A The misperceived social norm of drunkenness among early adolescents in Finland Health Educ Res 2004 19 1 64 70 10.1093/her/cyg010 15020546

[B29] Humeniuk RE Henry-Edwards S Ali RL Poznyak V Monteiro M The Alcohol, Smoking and Substance Involvement Screening Test (ASSIST): manual for use in primary care 2010 World Health Organisation, Geneva

[B30] Young AM Glover N Havens JR Nonmedical use of prescription medications among adolescents in the United States: A systematic review J Adolesc Health 2012 51 1 6 17 10.1016/j.jadohealth.2012.01.011 22727071

[B31] European Monitoring Centre for Drugs and Drug Addiction 2011 Annual report on the state of the drugs problem in Europe 2011 EMCDDA, Lisbon http://www.emcdda.europa.eu/publications/annual-report/2011

[B32] Core Institute Core Alcohol and Drug Survey Long Form 2008 University Carbondale, Southern Illinois

[B33] Bewick BM West R Gill J O'May F Mulhern B Barkham M Hill AJ Providing web-based feedback and social norms information to reduce student alcohol intake: A multisite investigation J Med Internet Res 2010 12 5 e59 10.2196/jmir.1461 21169171 PMC3057315

[B34] Neighbors C Dillard AJ Lewis MA Bergstrom RL Neil TA Normative misperceptions and temporal precedence of perceived norms and drinking J Stud Alcohol 2006 67 2 290 299 16562412 10.15288/jsa.2006.67.290 PMC2443635

[B35] West SG Finch JF Curran PJ Hoyle RH Structural equation models with nonnormal variables: Problems and remedies Structural equation modeling: Concepts, issues, and applications 1995 Sage, Thousand Oaks 56 75

[B36] Kline RB Principles and practice of structural equation modeling 1998 Guilford Press, New York

[B37] DeJong W The role of mass media campaigns in reducing high-risk drinking among college students J Stud Alcohol Suppl 2002 14 182 192 12022724 10.15288/jsas.2002.s14.182

[B38] Hastings G Stead M Webb J Fear appeals in social marketing: Strategic and ethical reasons for concern Psychology and Marketing 2004 21 11 961 986 10.1002/mar.20043

[B39] Foxcroft DR Ireland D Lister-Sharp DJ Lowe G Breen R Longer-term primary prevention for alcohol misuse in young people: a systematic review Addiction 2003 98 4 397 411 10.1046/j.1360-0443.2003.00355.x 12653810

[B40] Evans RI Matarazzo JD, Weiss SM, Herd JA, Miller NE, Weiss SM A social inoculation strategy to deter smoking in adolescents Behavioral health: A handbook of health enhancement and disease prevention 1984 Wiley, New York 765 774

